# Videos using different message strategies to promote the interruption of sedentary behavior in university students during online lectures – A pilot study

**DOI:** 10.3389/fpubh.2023.1108154

**Published:** 2023-07-27

**Authors:** Anastasia Doré, Kristin Kalo, Lisa Schwab, Jennifer L. Reichel, Laura Eisenbarth, Tilmann Strepp, Robin Jacob, Kira Enders, Stephan Letzel, Perikles Simon, Pavel Dietz, Thomas Kubiak, Sebastian Heller

**Affiliations:** ^1^Department of Health Psychology, Institute of Psychology, Johannes Gutenberg University Mainz, Mainz, Germany; ^2^Department of Sports Medicine, Disease Prevention and Rehabilitation, Johannes Gutenberg University Mainz, Mainz, Germany; ^3^Institute of Occupational, Social and Environmental Medicine, University Medical Centre of the University of Mainz, Mainz, Germany; ^4^Department of Sport and Exercise Science, University of Salzburg, Salzburg, Austria

**Keywords:** student health, setting-based health promotion, sedentary behavior, health communication, narrative evidence, vividness

## Abstract

**Background:**

Sedentary behavior (SB) is highly prevalent among university students and has increased during COVID-19 pandemic. As SB is associated with negative health outcomes, appropriate prevention measures in the university setting are needed.

**Objective:**

This pilot study aimed at investigating the effects of videos using different message strategies to interrupt SB in the collective of university students during online lectures.

**Methods:**

During online lectures, university students (*N* = 96) were shown one of three videos on the interruption of SB. The videos differed in their message strategies with regard to evidence type (statistical vs. narrative) and vividness (static vs. animated images). Demographics, health variables (SB intentions, SB attitudes) and selected media reception variables (identification, homophily, counterarguing) were examined as possible influence factors on the interruption of SB evoked by watching the video.

**Results:**

Approximately half of the students interrupted sedentary behavior during watching the videos and students of the older age group (cut-off: median = 22 years) interrupted SB significantly more often (*p* = 0.046). The interruption of SB was predicted by SB intentions (*p* < 0.05). Identification with characters significantly predicted the intentions to reduce SB (*p* < 0.001), with a large effect of the overall regression model (*R*^2^_corr_ = 0.47).

**Conclusion:**

Considering the increased digitalization in general and restrictions due to COVID-19 pandemic, videos seem to be a useful tool to interrupt SB among university students during online lectures. Narrative formats could facilitate the intention to reduce SB, which in turn could have a positive impact on the interruption of SB. However, further research on effective communication and message strategies is needed.

## 1. Introduction

Sedentary behavior (SB) is defined as any behavior in an awake state in sitting, leaned or horizontal position with an energy consumption less than 1.5 MET (metabolic equivalent) (1, 2). Populations of high-income countries spend a large extent of their daily time with SB (3). University students show an especially large extent of SB with sitting times of approximately 10 h per day (4, 5). Particularly common study activities, such as attending lectures, literature research, or writing assignments, are often spent in sedentary positions (4, 6, 7). Additionally, recent studies implicate that SB among university students further increased during the COVID-19 pandemic, whereas health beneficial physical activity simultaneously decreased (8, 9).

From a public health point of view, the above-mentioned extent of SB among university students and its increase during the COVID-19 pandemic are alarming because SB represents a clinically relevant risk factor which is associated with various physiological [e.g., (10–12)] and psychological [e.g., (7, 12, 13)] burdens and diseases, and may lead to an increased mortality [e.g., (11, 14)]. Therefore, effective measures to reduce SB in the university setting are needed. In this context, research suggests that not only the overall reduction of time spent with SB, but also regular interruptions of SB may be beneficial (6, 15) – even with short bouts of light physical activity (bodily movements produced by skeletal muscles that require energy expenditure of less than 3 MET, e.g., walking slowly or low impact exercises like light stretching) (16). The adaption of environmental conditions such as standing desks (17, 18) represents one commonly used option to regularly interrupt SB with light physical activity, but it may be limited due to financial restrictions and environmental constraints pertaining to workplace design and study conditions at home (6). Therefore, behavioral prevention approaches of health communication at the target group level of university students may be a promising approach to tackle the large extent of SB (6, 19).

Health communication aims to facilitate positive health outcomes by improving communication processes (20, 21). Among various distribution methods of health communication, especially mass media-based health communication is used in current times of increasing digitalization and it includes communication via different channels, such as (online) brochures and videos. In general, health communication uses different strategies, which refer to the overall approach to change health relevant parameters and can be organized in cognitive, affective, social, and behavioral categories (22). In particular, message strategies include clearly defined and manipulable characteristics of a message, which can have different effects on recipients’ health outcomes (23).

One popular message strategy in health communication is the use of different evidence types – either statistical or narrative evidence. Statistical evidence is usually based on a large number of cases to disseminate information and appeals to the ratio of recipients (24–26). By contrast, within narrative evidence, cohesive stories with one or more protagonists are told (25, 26). Theories of narrative health communication assume that processes such as homophily and identification with media characters are effective mechanisms to reduce resistance against persuasive messages (such as counterarguing) and influence health behavior by changing health attitudes and intentions [e.g., (27, 28)]. Whereas homophily describes a cognitive evaluation of perceived similarity with a character (27), identification refers to a deeper process of adopting the perspective of a character (28). A recent meta-analysis indicates the effectiveness of both evidence types, but it remains unclear which strategy is more successful among which target group and in which setting (26).

Another message strategy used within health communication is the level of vividness. Vivid communication is conceptualized as “likely to attract and hold our attention and to excite the imagination to the extent that it is emotionally interesting, concrete and imagery-provoking, and proximate in a sensory, temporal, or spatial way” [(29, p.45)]. In literature, vividness has been manipulated in different ways, including the presence or absence of pictures and concrete or abstract pictures (30). A meta-analysis suggests that vivid information is more persuasive than non-vivid information (30). Concurrently, the *cognitive load theory of multimedia learning* assumes that the working memory of individuals is limited with separate channels for visual/pictorial and auditory/verbal material (31). Thus, the theory states that learning-irrelevant cues such as the use of too many different colors or a noisy voice-over should be reduced.

A systematic review of 28 studies suggests the effectiveness of video-based communication in modifying health behavior in general (32). Targeting university with video-based health communication, Conceição et al. (33) found an increase of fertility knowledge and in another study Conceição et al. (34) observed a reduction of depression stigma and an increase in help-seeking attitudes. In addition, a recent study indicated that videos are as effective as text formats in changing meningitis B vaccine knowledge, perceptions, and intentions (35). Particularly, when considering the increasing digitalization in general and the restrictions of regular university teaching caused by the COVID-19 pandemic, videos applied during online lectures seem like a promising approach to communicate health information to university students. However, evidence about the effects of different message strategies to promote the interruption of SB is lacking, particularly in the setting of online lecturing. Furthermore, it remains unclear, which characteristics of the heterogeneous target group of students (e.g., age or gender) are associated with the potential effects of these interventions. To determine the effectiveness of health communication interventions, it is further crucial to include psychological variables such as health intentions and attitudes in the investigation, as common theories, such as the *theory of planned behavior*, emphasize the relevance of these processes for the adaption of health behavior (36, 37).

Therefore, the present pilot study aimed at (1) identifying potential differences in the prevalence of the interruption of SB between video formats using different message strategies (evidence type, vividness), gender, and age groups. (2) In a binary logistic regression analysis, it was further investigated, if the interruption of SB was predicted by the intention to reduce SB, the attitude towards SB, age, and gender. (3) Finally, leaning on theories about narrative persuasion (27, 38), it was examined if the intention to reduce SB and the attitude towards SB were predicted by identification and homophily with characters, as well as counterarguing.

## 2. Materials and methods

### 2.1. Study design

A three-arm parallel grouped study design was used to examine the effects of different video formats on the interruption of SB during online lectures. The study was conducted as part of the ongoing project “Healthy Campus Mainz” which aims at building and evaluating an evidence-based student health management program at the Johannes Gutenberg-University Mainz (JGU). For this purpose, the expertise of various professions (medicine, psychology, sports science, media science) is combined. Further information about the project is provided by Reichel et al. (39). The study was approved by the Ethics committee of the federal state of Rhineland-Palatinate (Number: 2021-15876-other research) and followed the Declaration of Helsinki Ethical Principles for Medical Research Involving Human Subjects. Participants received no compensation.

### 2.2. Participants and procedure

Student participants were recruited *via* lectures in different disciplines (e.g., psychology, sports science, medicine) at the Johannes Gutenberg University (JGU) Mainz. Through personal contact, 23 lecturers were approached who enlisted further lecturers through word-of-mouth recruitment. Overall, the videos were shown in 16 lectures, which were divided into small and large ones to ensure equivalent sizes of the experimental groups. For this purpose, *n* = 50 was set as a cut-off to separate small practical seminars from large lectures. A cluster randomization subsequently was performed counter-balanced and separately for small and large lectures using BiAS for Windows version 11.10 (40). Courses were randomized to three experimental groups, each of them watching the video in one of three video formats (animated-statistical, animated-narrative, static-statistical) during online lectures. Videos were either incorporated in asynchronous lectures (e.g., pre-recorded lectures, on demand) or in synchronous lecture formats such as video conferences. After watching the videos, participants consented study participation and completed an online survey containing questionnaires on demographic, health, and media reception variables.

### 2.3. Video intervention

Since various evidence identified SB as risk factor in university students [e.g., (4, 5)], the “Health Express” has been developed as an intervention for the communication of health-promoting information and behaviors. The “Health Express” is a video-based intervention consisting of videos with a duration of approximately 3 min, which are embedded into online lectures at the JGU. It aims at imparting evidence-based knowledge of SB and gives information about prevention strategies of SB in students’ everyday life. The videos present health-promoting information in three different video formats, which vary in their message strategy using different evidence types (statistical vs. narrative) and level of vividness (static vs. animated). Please find the video links listed in (Supplementary Table 1).

Two video formats (animated-statistical, animated-narrative) are presented as animated videos using the same moving images depicting two student characters but with the voice-over in different evidence types. These video formats were developed by the study team using the online platform Powtoon (41). The animated-statistical video format uses statistical evidence by presenting information on a large number of individuals [e.g., number of JGU students fulfilling the World Health Organization’s (WHO) recommendations for physical activity (42)]. By contrast, in the animated-narrative video format, the same information is presented in a narrative form: a dialog between the depicted university students is presented, one person describing barriers of reducing SB and the other person identifying possible solutions. No further information about the characters is given to facilitate identification for a wide majority of university students. As students are exposed to numerous visual and auditory cues during online lectures, it could also be suitable to use health communication with a low level of vividness. Therefore, the static-statistical video format (in contrast to the two other video formats) includes a lower level of vividness by using no moving images, and information in the style of statistical evidence is given through professional speakers.

### 2.4. Measures

The survey included questionnaires measuring demographics, health variables (interruption of SB, intention to reduce SB, attitude towards SB), and media reception variables (identification with characters, homophily with characters, counterarguing).

#### 2.4.1. Demographics

Gender, semester, and subject were measured by means of categorical variables. As only few students reported to study another subject than psychology, medicine, or sports science, we summarized the remaining subjects to the category other. The mean and the standard deviation for semester and the continuous variable age were calculated. Additionally, we dichotomized age by means of the median (22 years) to be able to compare the age groups in the following analyses.

#### 2.4.2. Health variables

The following single item was used to measure the interruption of SB: “Did you stand up during watching the video?” The answer format was binary (“Yes”/“No”).

To measure the intention to reduce SB, the three items of Chevance et al. (43) were adapted to SB and translated into German [“I intend to reduce (my) sedentary behavior after watching the video.”; “Even if I am tired, alone, or sick, I have set the objective of limiting my sedentary behavior after watching the video”; “Even if I have to work a lot for my study, I will limit my sedentary behavior after watching the video”]. The answer format was a 7-point Likert scale (from “I absolutely agree” to “I absolutely disagree”). Cronbach’s alpha was *α* = 0.85.

The questionnaire to measure the attitude towards SB was developed based on various studies (44–46), and translated into German. Within this scale, which included 3 items, the first was: “Reducing sedentary behavior would make me…” “…very satisfied” to “very dissatisfied.” The remaining two items [e.g., “I consider the reduction of sedentary behavior as beneficial for my health.,” and “It is not important for me to reduce my sedentary behavior (inversed)”] included a 5-point Likert scale answer format (from “I agree very much” to “I disagree very much”). Cronbach’s alpha was *α* = 0.36.

#### 2.4.3. Media reception variables

To measure identification with characters, *Cohen’s identification scale* (28) was used and translated into German. An exemplar item is: “During watching, I felt I could really get inside the characters’ head.” The answer format was a 5-point Likert scale (from “I agree very much” to “I disagree very much”). We measured a Cronbach’s alpha of *α* = 0.84.

Perceived homophily with characters was captured through a translated version of the dimension *attitude* of the *Attitude homophily scale* (47) (e.g., “The characters…” “… behave like me” to “…do not behave like me”). Cronbach’s alpha for this scale was *α* = 0.84 which is in line with reliabilities other studies established for this scale (48).

To measure counterarguing, the 4 items of the study of Nabi et al. (49) were translated into German. The answer format was a 5-point Likert scale (from “I agree” to “I disagree at all”). Nabi et al. (49) found a single factor index with a Cronbach’s alpha of *α* = 0.80.

### 2.5. Data analysis

Statistical requirements for every test were checked before analysis. Chi^2^-tests were conducted to investigate differences in the prevalence of interruptions of SB between video formats, gender, and age. To investigate if the interruption of SB was predicted by the intention to reduce SB, attitude towards SB, gender, and age, a binary logistic regression was computed. If attitude towards SB, and intention to reduce SB appeared to be significant predictors in this model, the prediction of these variables was analyzed in a further step using two multiple linear regressions with identification with characters, homophily with characters, and counterarguing as predictors. For this purpose, only animated formats were involved into calculations. Analyses were conducted using IBM SPSS (50).

## 3. Results

Overall, *N* = 118 university students participated in the study. Of these participants, *n* = 22 indicated that they have already seen the video in another lecture or provided no data or only demographic data and thus were not considered in the analyses, resulting in a final sample size of *N* = 96 (animated-statistical group: *n* = 29, animated-narrative group: *n* = 32, and static-statistical group: *n* = 35). Participants were on average 23.54 years old (*SD* = 3.93), and more than half of the sample identified as female (62.5%). In Table 1, a description of the study sample in total and subdivided by video format is provided. The most common subject was psychology (45.0%). On average, participants studied in semester 4.77 (*SD* = 2.46), with 12% being in their first or second semester. Experimental groups did not significantly differ in gender [χ^2^ (2) = 0.946, *p* = 0.623, *V* = 0.623] or semester [*F* (2, 91) = 2.139, *p* = 0.124], but in study subject [χ^2^ (6) = 67.816, *p* < 0.001, *V* = 0.000] and age group [χ^2^ (2) = 15.568, *p* < 0.001, *V* = 0.000]. Table 2 presents descriptive data for the experimental variables, separately for gender and age groups.

**Table 1:** Demographic characteristics of the study sample.

Baseline characteristic	Animated-statistical		Animated-narrative		Static-statistical		Overall	
	*n*	%	*n*	%	*n*	%	*N*	%
Gender								
Male	9	31	12	37.5	15	43	36	37.5
Female	20	69	20	62.5	20	57	60	62.5
Study subject								
Psychology	16	55	7	22	20	57	43	45
Sport science			13	41	15	43	28	29
Medicine	13	45					13	14
Other			10	31			10	10

	*N*	*M*	*SD*	*N*	*M*	*SD*	*N*	*M*	*SD*	*N*	*M*	*SD*
Age	29	25.0	2.90	32	23.1	4.21	34	22.7	4.16	95	23.5	3.93
Semester	29	5.52	1.82	30	4.6	3.34	35	4.29	1.89	94	4.77	2.46

*n* = absolute frequency; % = relative frequency.

**Table 2:** Descriptive statistics of experimental variables.

Variable	Overall			Female			Male			Chi^2^	≤ 22 years			> 22 years			Chi^2^
	*N*	*M*	*SD*	*N*	*M*	*SD*	*N*	*M*	*SD*	*p*	*N*	*M*	*SD*	*N*	*M*	*SD*	*p*
Identification	96	2.4	0.57	60	2.3	0.51	36	2.5	0.65	0.415	50	2.5	0.59	45	2.3	0.51	0.430
Homophily	95	3.5	1.12	60	3.3	1.04	35	3.7	1.23	0.434	49	3.6	1.17	45	3.3	1.04	0.989
Counterarguing	93	4.0	0.53	59	4.1	0.49	34	3.8	0.58	0.029*	47	4.0	0.59	45	4.01	0.48	0.879
Intention	92	3.1	1.00	59	3.1	0.87	33	3.2	1.21	0.567	47	3.3	1.02	44	2.9	0.96	0.222
Attitude	92	2.0	0.55	59	1.9	0.49	33	2.1	0.65	0.466	47	2.1	0.54	44	1.9	0.54	0.316

*p < 0.05.

### 3.1. Differences in interrupting sedentary behavior between video formats and demographic variables

Of all participants, 51% (*n* = 47) interrupted SB while watching the video, whereas 49% (*n* = 45) indicated that they did not interrupt SB. The prevalence of interruption of SB was 36% (*n* = 12) in the static-statistical group, 57% (*n* = 16) in the animated-statistical group, and 61% (*n* = 19) in the animated-narrative group. On a descriptive level, more men (61%, *n* = 20) than women (46%, *n* = 27) interrupted SB. However, there was no significant difference between gender [χ^2^ (1) = 4.565, *p* = 0.172, *V* = 0.172] or video format [χ^2^ (2) = 4.565, *p* = 0.102, *V* = 0.102] in the interruption of SB. By contrast, age groups significantly differed in the interruption of SB [χ^2^ (1) = 3.986, *p* = 0.046, *V* = 0.046], with more students of the older age group (62%, *n* = 27) interrupting SB while watching the video, compared to the younger students (40%, *n* = 19).

### 3.2. Prediction of the interruption of sedentary behavior

The binary logistic regression model to predict the interruption of SB by the variables intention to reduce SB, attitude towards SB, gender, and age was statistically significant [χ^2^ (4) = 14.071, *p* = 0.007] and explained 19% of the variance (Table 3), indicating a small effect (51). Intention to reduce SB significantly predicted the interruption of SB (*p* < 0.05), while attitude towards SB (*p* = 0.762), gender (*p* = 0.158), and age (*p* = 0.099) were no significant predictors in this model.

**Table 3:** Prediction of the interruption of sedentary behavior.

Variable	*B*	*SE*	Wald	*p*	Exp (*B*)	95% *CI*
Intention	0.598	0.277	4.658	0.031*	1.818	[1.056–3.310]
Attitude	0.138	0.457	0.092	0.762	1.148	[0.469–2.812]
Age	−0.107	0.065	2.727	0.099	0.898	[0.791–1.020]
Gender	0.702	0.496	1.998	0.158	2.017	[0.762–5.338]

Chi^2^: *χ*^2^ (4) = 14.071, *p* = 0.007. Model fit: *R*^2^ = 0.191 (Nagelkerke). 95% *CI* for Exp (*B*).*p < 0.05.

### 3.3. Prediction of the intention to reduce sedentary behavior

As attitude towards SB was no significant predictor for the interruption of SB, regression analysis was only calculated with intention to reduce SB as the dependent variable. The included predictors of the model were identification and homophily with characters, as well as counterarguing. The regression model was significant [*F* (3, 52) = 18.172, *p* < 0.001] with a corrected *R*^2^ of 47%, indicating a large effect (52). Identification was a significant predictor of the model (*p* < 0.001), whereas homophily (*p* = 0.973), and counterarguing (*p* = 0.397) did not significantly predict the intention to reduce SB (Table 4).

**Table 4:** Prediction of the intention to reduce sedentary behavior.

Variable	*B*	*SE*	*Β*	*T*	*p*	95% *CI*
Identification	1.260	0.158	0.689	6.360	0.000**	[0.863–1.656]
Homophily	−0.003	0.102	−0.004	−0.034	0.973	[−0.208–0.210]
Counterarguing	−0.161	0.188	−0.083	−0.855	0.397	[−0.538–0.217]

ANOVA: *F* (3, 55) = 18.172, *p* < 0.001. Model fit: *R*^2^ = 0.470 (corrected). 95% *CI* for *B*.**p < 0.001.

## 4. Discussion

Videos seem to be an appropriate measure of health communication to interrupt SB in university students during online lectures, since overall, approximately half of the participants stated that they interrupted SB during watching the video. The interruption of SB differed between age groups, but not between gender or video formats. The interruption of SB is predictable by a binary logistic regression, with intention to reduce SB as the only significant predictor of the model. The intention to reduce SB was in turn determined by identification with characters, and the model showed a high level of explained variance.

Referring to the first research aim, namely to identify potential group differences in the prevalence of the interruption of SB, age has been identified as relevant factor for the prevalence of SB (53–55). In general, SB seems to increase with growing age, but with exception for the SB of computer use (54, 55). Regarding the student population, Deliens et al. (56) found that “freshmen” showed more SB, what is congruent with the current finding that younger students significantly less often interrupted SB. One explanation is that health risk behavior is more prevalent in younger adults and reinforced by the loss of parental control in the transitional phase of study begin (56). Furthermore, other research indicates that younger adults score higher at trait reactance evoked by the insecurity of the own autonomy (57), what possibly explains the resistance towards the promoted behavior within the video.

Although findings of our study showed no significant difference between video formats, descriptive data indicated a tendency towards animated video formats being more effective to promote the interruption of SB. This preliminary finding corroborates the findings of a meta-analysis, which found vivid information to be more persuasive than messages low in vividness (30). In addition, vividness of health communication interventions and identification with characters showed to be correlated (58), what hints to the effectiveness of using moving images presenting characters who facilitate identification. Another possible explanation for this preliminary result is that animated images may be more able to gain attention during online lectures which are demanding for students’ concentration and additionally may be suitable for a topic requiring being active. Depicting possible differences between narrative and non-narrative video formats, the current results are incongruent with a recent study (59) which found that narrative videos were superior to non-narrative videos in reducing students’ subsequent sedentary behavior while playing video games. In our study, narrative and non-narrative formats only varied with regard to the application of a dialog, but the same moving images were presented. As we did not conduct a manipulation check, potentially participants did not perceive the formats differently with regard to the extent of narrative elements and level of identification and no significant differences between formats could be observed.

With regard to the second research aim, namely to investigate if the interruption of SB was predicted by the intention to reduce SB, the attitude towards SB, age, and gender, a significant association between SB intentions and the interruption of SB was illustrated, which supports other study results (53, 60, 61). Prapavessis et al. (61) found that attitude predicted intention, what in turn determined the reduction of SB. Similarly, our study did not indicate a direct link between attitude and SB interruption, but a significant correlation between intention and attitude (*p* = 0.343, *p* < 0.001). This result is congruent with the *theory of planned behavior*, which states that attitude affects behavior indirectly through a correlation with intention, which subsequently influences health behavior (36).

In view of the third research aim, to examine if the intention to reduce SB and the attitude towards SB was predicted by identification and homophily with characters, as well as counterarguing, identification with characters was the only significant predictor of intention to reduce SB. Attitude towards SB was not predicted because it was no significant predictor of the interruption of SB in our analysis. With regard to these results, different studies that investigated the correlation between identification with media characters and health intentions indicated mixed results for different health behaviors. Whereas the study of Murphy et al. (62) did not find an association between the identification with characters of a narrative video and intentions to have a Pap test for cancer prevention, other research supports our finding that identification with characters of narrative health interventions predict health intentions (58, 63). In this context, results of Moran et al. (64) point to an indirect effect of identification on health intentions *via* social norms, although contrary to the hypothesis, the association between identification and social norms was negative. By contrast, other studies found a direct significant association between the identification and health intentions (58, 65). Dillard and Main (58) observed a correlation with identification for intentions to have a colonoscopy and Moyer-Gusé et al. (65) for intentions to discuss sexual-transmitted diseases (STIs). In doing so, Moyer-Gusé et al. (65) emphasize the relevance of self-efficacy for the link between identification and health intentions. A recent meta-analysis investigating the effects of celebrity health communication supports the positive association between identification and health intentions (63). In general, narrative evidence interventions seem to be more effective to change health intentions, whereas statistical evidence interventions have a greater impact on health attitudes (26). Zebregs et al. (26) concluded that intentions involve a greater affective component than attitudes, thus emphasizing the effectiveness of increasing identification with characters to strengthen persuasion.

The current study has limitations. Some of the used measures were not validated, thus representing a possible source of bias. Results showed that Cronbach’s alpha of the scale attitude towards SB was low, indicating an inappropriate measurement of this variable. As this is a pilot intervention, we conducted no power analysis to calculate the sample size, and thus the sample size could have been too small. Further, comprised university students mostly studied a health-related subject (psychology, sports science, medicine) what limits generalization of the findings. However, existing research suggests that the amount of SB is not linearly associated with moderate-to-vigorous-intensity physical activity (66). In fact, the extent of light-intensity physical activity (e.g., walking to the campus, to the bus station, or between lectures) and SB are inversely correlated (67), which indicates that everyday life physical activity might be more important to regularly interrupt SB than moderate-to-vigorous intensities of physical activity which predominantly occur in exercising or engaging in sports (68). As the investigation did not include a passive control group, the general effect of video-based interventions to promote the interruption of SB during online lectures could not be determined. Additionally, only the behavioral short-term effects on the interruption of SB were depicted and not the impact on general SB.

Future studies should ensure an appropriate sample size, an equal distribution of participants across all study subjects, as well as an investigation of long-term effects on SB, and an inclusion of a passive control group. Based on the current findings and theoretical approaches (36), future studies could analyze if SB intentions mediate the correlation between SB attitudes and SB behavior. In this context, it is important to include an objective measure for SB behavior and to develop and validate appropriate questionnaires of SB related constructs, particularly the attitude towards SB. Additionally, prospective studies could include other factors that might be relevant to the effectiveness of video-based interventions, such as physical acitvity habits (69), stress (70) as well as enviromental and social determinants (56, 71, 72). In general, future research should investigate different message strategies to promote the interruption and reduction of SB in university students. In doing so, placing a focus on the channel video and an application during online lectures could be useful. Based on the current results, future studies could consider possible differences in media reception variables such as counterarguing when examining narrative video formats. The acceptance and the feasibility of health communication interventions to reduce SB should also be analyzed among university lecturers to facilitate the implementation into (online) lectures. Within the use of narrative evidence to communicate health information, message strategies such as the use of animated vs. real characters could be examined. In this context, a recent study suggests that students increased their physical activity in response to influencers on Instagram (73). Thus, influencers and relatable testimonials could be one opportunity to strengthen identification, increase SB intentions and reduce SB in college students. From a broader perspective, future research should investigate the effectiveness of health communication interventions for different health topics in the collective of university students, including the use of different message strategies and channels.

### 4.1. Conclusion and practice implications

Using a unique approach to compare different video formats for health communication during online lectures, this pilot study implies the suitability of short videos to interrupt SB during online lectures in the target group of university students. In doing so, the current results highlight the importance of tailoring these videos to the characteristics of the target group, particularly considering the age of the target group. When using narrative formats, identification with characters should be increased to facilitate the intention to reduce SB which in turn could have a positive impact on the interruption of SB. In particular, identification with videos’ characters could be strengthen by using appealing characters who are similar to the student population regarding demographic, social and environmental factors. In this context, the use of a high level of vividness, e.g., by using moving images, could enhance the process of identification. In line with other research (74, 75), the videos could also be presented in other university settings and contexts, such as in face-to-face lectures, public university spaces or as reminders on university websites or via e-mail. Further research is needed to get more insights into relevant mechanisms and effective message strategies of video-based health communication interventions to reduce SB in university students as well as applicability to different university settings.

## Data availability statement

The raw data supporting the conclusions of this article will be made available by the authors, without undue reservation.

## Ethics statement

The studies involving human participants were reviewed and approved by the Ethics committee of the federal state of Rhineland-Palatinate (Number: 2021-15876-other research). The patients/participants provided their written informed consent to participate in this study.

## Author contributions

AD, KK, LS, JR, LE, TS, RJ, KE, PD, TK, and SH: conception and design. AD, KK, LS, JR, RJ, KE, PD, TK, and SH: acquisition of the data. AD, KK, PD, TK, and SH: analysis of the data and drafting of the article. AD, KK, LS, JR, LE, TS, RJ, KE, SL, PS, PD, TK, and SH: critical revision of the article for important intellectual content and final approval of the article. All authors contributed to the article and approved the submitted version.

## Funding

The Healthy Campus Mainz project was funded by BARMER health insurance and carried out with the support of the Johannes Gutenberg-University of Mainz and the University Medical Center of the Johannes Gutenberg-University of Mainz.

## Conflict of interest

The authors declare that the research was conducted in the absence of any commercial or financial relationships that could be construed as a potential conflict of interest.

## Publisher’s note

All claims expressed in this article are solely those of the authors and do not necessarily represent those of their affiliated organizations, or those of the publisher, the editors and the reviewers. Any product that may be evaluated in this article, or claim that may be made by its manufacturer, is not guaranteed or endorsed by the publisher.
