# Supplementary material for: Videos using different message strategies to promote the interruption of sedentary behavior in university students during online lectures – A pilot study
Source: Front Public Health. 2023 Jul 27;11:1108154. doi: 10.3389/fpubh.2023.1108154 (PMC10412984; doi:10.3389/fpubh.2023.1108154)
Supplement: Supplementary file 1 [file Table_1.pdf]

Supplementary Material

Videos using different message strategies to promote the interruption of sedentary behavior in university students during online lectures – A pilot study

Anastasia Doré[1], Kristin Kalo[2], Lisa Schwab[2], Jennifer L. Reichel[3], Laura Eisenbarth[3], Tilmann Strepp[4], Robin Jacob[3], Kira Enders[2], Stephan Letzel[3], Perikles Simon[2], Pavel Dietz[3], Thomas Kubiak[1], Sebastian Heller[3*]

[1]Department of Health Psychology, Institute of Psychology, Johannes Gutenberg University Mainz, Mainz, Germany

[2]Department of Sports Medicine, Disease Prevention and Rehabilitation, Johannes Gutenberg University Mainz, Mainz, Germany

[3]Institute of Occupational, Social and Environmental Medicine, University Medical Centre of the University of Mainz, Mainz, Germany

[4]Department of Sport and Exercise Science, University of Salzburg, Salzburg, Austria

*** Correspondence:** Corresponding Author: seheller@uni-mainz.de

Supplementary Table 1. Links to the videos used in the Health Express pilot study

Video format	Link to the video
Animated-narrative video	https://video.uni-mainz.de/Panopto/Pages/Viewer.aspx?id=99e98a60-d4f1-4d1a-a46c-af5800f512fd
Animated-statistical video	https://video.uni-mainz.de/Panopto/Pages/Viewer.aspx?id=5ad4ab2d-d167-49c8-9733-af5800f52fcb
Static-statistical video	https://video.uni-mainz.de/Panopto/Pages/Viewer.aspx?id=0dc8c759-22f0-4f06-ab1c-af5800f541fc
